# Biochemical Profiling and Ecotoxicological Assessment of *Prunus spinosa* L. Fruits from the Cluj Region: Phytochemical Composition, Contaminant Load, and Antioxidant Potential

**DOI:** 10.3390/foods15173135

**Published:** 2026-09-03

**Authors:** Vanda Băbălău-Fuss, Lucian Dordai, Marius Roman, Socaciu Bianca, Traian Ciprian Stroe, Adrian Vasile Timar, Anca Becze

**Affiliations:** 1 National Institute for Research and Development of Optoelectronics INOE 2000, Research Institute for Analytical Instrumentation, 67 Donath Street, 400293 Cluj-Napoca, Romania; vanda.fuss@icia.ro (V.B.-F.); marius.roman@icia.ro (M.R.); bianca.socaciu@icia.ro (S.B.); anca.naghiu@icia.ro (A.B.); 2 Department of Natural Sciences, Faculty of Natural and Agricultural Sciences, Ovidius University of Constanța, University Alley, 1, Campus Building B, 900470 Constanta, Romania; str_ciprian@yahoo.com; 3 Department of Environmental Engineering, Faculty of Environmental Protection, University of Oradea, 410048 Oradea, Romania; atimar@uoradea.ro

**Keywords:** *Prunus spinosa*, blackthorn, medicinal plants, food additives, antioxidant capacity, polyphenols, PAHs, heavy metals, ecotoxicological assessment, PCA, Cluj region

## Abstract

*Prunus spinosa* L. (blackthorn) is a widely distributed wild fruit species in Romania with considerable, yet underexplored, potential as a functional food ingredient and natural source of bioactive compounds. This study aimed to characterize the phytochemical composition, mineral and contaminant profiles, and antioxidant capacity of *P. spinosa* fruits collected from five sites in the Cluj region with contrasting land-use conditions (forest-edge, rural, suburban, and roadside). Fruits were harvested in three sampling campaigns (October 2018, October 2019, and November 2019). Analytical determinations included total polyphenol content, antioxidant capacity, fatty acid methyl esters (FAMEs), dietary fibers, chlorophyll, water content, polycyclic aromatic hydrocarbons (PAHs, 15 congeners), and metal concentrations (ICP-OES/MS). A composite Ecotoxic Pressure Index (EPI) integrating metal and PAH data was computed per site. Total polyphenol content ranged from 124 to 1172 mg GAE·kg^−1^ DW, while antioxidant capacity ranged from 116 and 1184 µg AAE·mg^−1^. One-way ANOVA revealed significant location effects on Cu, Zn, Mn, Ca, Mg, Na and K (*p* < 0.05). PCA (65–70% explained variance) discriminated rural/forest-edge samples (high polyphenols, low EPI) from urban/roadside samples (elevated metals and PAHs). Multiple linear regression demonstrated that polyphenols were the dominant positive predictor of antioxidant capacity (β = +0.78, *p* < 0.001), while Fe and total PAHs were inversely associated with antioxidant capacity (β = −0.31 and −0.28, respectively; R^2^ = 0.80). The observed biochemical and contaminant profiles varied according to geographical origin and land-use context, although these associations cannot be attributed exclusively to pollution exposure. Rural and forest-edge samples showed favorable descriptive profiles for several bioactive parameters, but further controlled studies are required before site-specific superiority for functional food applications can be established.

## 1. Introduction

Wild edible and medicinal plants constitute a renewable source of bioactive compounds with well-documented relevance to human health, food technology, and environmental monitoring [1,2]. Romania hosts a highly diverse flora, comprising over 3600 vascular plant species, of which more than 10% are traditionally used in phytomedicine [3]. Among these, *Prunus spinosa* L. (blackthorn) is a widespread shrub of the *Rosaceae* family, commonly found along forest margins, hedgerows, and semi-natural landscapes. Despite its long-standing ethnopharmacological use, the comprehensive biochemical and environmental characterization of Romanian *P. spinosa* populations remains limited [4,5].

The fruits of *P. spinosa* are recognised for their rich composition in biologically active compounds, including vitamin C (ascorbic acid), vitamin E (α-tocopherol), B-complex vitamins, flavonoids, tannins, catechins, organic acids, pectins, coumarins, and unsaturated fatty acids [6,7]. From a food technology perspective, these constituents correspond to key functional additive categories, including natural antioxidants, acidity regulators, stabilisers, emulsifiers, colorants, and flavour enhancers [8]. *Prunus spinosa* has attracted increasing interest as a potential source of clean-label, plant-derived ingredients capable of replacing synthetic food additives [9].

The quality and safety of wild edible fruits are strongly influenced by environmental conditions, particularly in areas exposed to anthropogenic pollution [10]. Plants growing in roadside and peri-urban environments may accumulate polycyclic aromatic hydrocarbons (PAHs) originating from incomplete combustion processes, as well as trace metals (e.g., Cu, Zn, Ni, Mn, Fe) derived from traffic emissions and soil contamination [11]. Several PAHs, including benzo[a]pyrene, are classified as carcinogenic compounds, and their presence in food matrices is strictly regulated at the European level [12]. The use of toxic equivalency factors (TEFs) allows the estimation of the cumulative carcinogenic potential of PAH mixtures expressed as benzo[a]pyrene equivalents [13]. These contaminants not only raise food safety concerns but may also interfere with plant metabolic processes, potentially affecting the biosynthesis and stability of bioactive compounds [14]. This is particularly relevant for the Cluj metropolitan region, one of Romania’s major urban centres, where contrasting land-use types, from industrial periphery to forested margins, create a well-defined anthropogenic gradient [15].

Despite growing interest in *P. spinosa*, existing studies have typically focused on isolated aspects of its composition, such as phenolic content or antioxidant activity [6,7,16,17]. To date, no study has simultaneously integrated comprehensive phytochemical profiling, mineral composition, and PAH contamination within a unified multivariate framework to evaluate the combined influence of environmental gradients on fruit quality [18,19]. Furthermore, no composite ecotoxicological indicator has been applied to this species for comparative assessment of contamination levels across collection sites, and the relationship between environmental pollutant load and antioxidant capacity has not been quantitatively modelled for wild *Prunus* taxa in the Carpathian region. This lack of integrative approaches represents a critical knowledge gap at the interface of food quality, safety, and environmental assessment.

Beyond the descriptive assessment of a regionally underutilised wild fruit, this study provides a comprehensive multi-analytical characterization of *P. spinosa* fruits as a potential food and functional-food resource. The study integrates phytochemical, biochemical, nutritional, mineral, and contaminant-related parameters to provide an integrated assessment of fruit composition, quality, antioxidant potential, and food-safety-related characteristics. In this context, the composite Ecotoxic Pressure Index (EPI) provides an integrative measure of multi-contaminant burden that complements the assessment of the nutritional and bioactive properties of the fruit. These findings contribute to the valorisation of *P. spinosa* as a potential functional food ingredient [20,21], while highlighting the importance of considering environmental context when evaluating the quality and food-safety aspects of wild-collected plant resources.

The present study addresses this gap through a multi-site investigation of *P. spinosa* fruits collected across different land-use contexts in the Cluj metropolitan region (Romania). The objectives of this work were: (i) to provide a comprehensive analytical characterisation of fruit composition, including polyphenols, antioxidant capacity, fatty acid profiles, mineral elements [22,23], and PAHs [24]; (ii) to develop and apply an Ecotoxic Pressure Index (EPI) as an integrative indicator of contaminant burden relevant to fruit quality and food-safety assessment; and (iii) to evaluate, using principal component analysis (PCA) and multiple linear regression, the relationships between environmental context, fruit composition, antioxidant-related quality parameters, and contaminant profiles.

## 2. Materials and Methods

**Figure 1:**
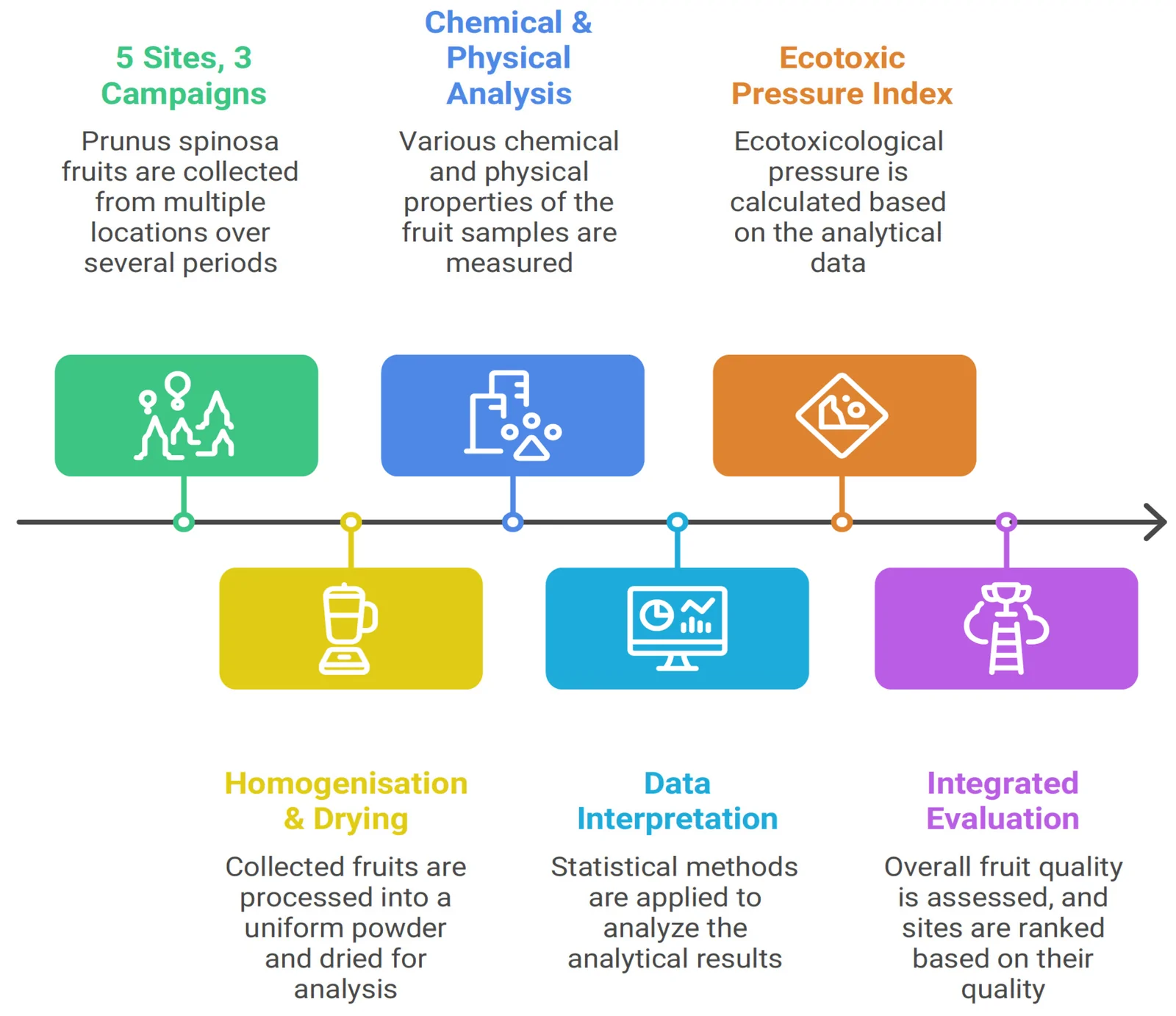
Overall experimental and analytical workflow for the biochemical and ecotoxicological assessment of *Prunus spinosa* fruits and extracts.

The overall experimental design and workflow of the study, spanning from raw material characterization to the comparative analysis of potential applications, is summarized in Figure 1.

### 2.1. Sampling Design and Study Sites

Fruit samples were collected across three temporal campaigns: Campaign 1 (October 2018), Campaign 2 (October 2019), and Campaign 3 (November 2019). A total of 40 composite batches were collected (20, 10, and 10 batches per campaign, respectively). Within each sampling site, *Prunus spinosa* shrubs were selected randomly from the naturally occurring population to minimize selection bias and ensure representative coverage of the local population. Fruits were collected from multiple randomly selected shrubs distributed throughout the sampling area rather than from a single individual plant. Only visually intact and mature fruits were harvested. Fruits collected from the selected shrubs within each site and sampling campaign were pooled to obtain composite batches representative of the respective location. The same sampling approach was applied across all five locations and sampling campaigns. Technical replicates belonging to the same composite batch were averaged before statistical analysis. Consequently, the 40 composite batches, not the individual technical measurements, were considered the independent experimental units.

All campaigns were conducted under favorable meteorological conditions at ambient temperatures of 6–8 °C. The five study sites, Borhanci, Chinteni, Făget, Florești, and Pata, were selected to represent contrasting land-use categories along an anthropogenic pollution gradient: background forest-edge (Făget), rural-agricultural (Chinteni, Borhanci), suburban-residential (Florești), and roadside/peri-industrial (Pata).

Potential outliers were screened separately for each variable using the Q1 − 3 × IQR and Q3 + 3 × IQR criterion. No observations exceeded these thresholds; therefore, no data points were excluded from the statistical analyses.

All analytical determinations for a given composite batch were performed using aliquots taken from the same homogenized sample material. The number of technical replicates varied among analytical techniques according to method-specific requirements, ranging from one to three measurements per batch. Where multiple technical replicates were available, their results were averaged within each batch and analytical variable to obtain a single batch-level value for each parameter. Thus, each composite batch contributed a single value per analytical variable to the statistical and multivariate analyses, avoiding differential weighting of variables according to the number of technical replicate measurements.

Following collection, fruit samples were sealed in polyethylene bags and transported to the laboratory under refrigerated conditions. Upon arrival, samples were homogenised, cleared of extraneous leaves and debris, and subjected to immediate water-content determination. Results are expressed on a dry-weight (DW) basis unless otherwise stated. A full list of matrices, parameters, and applied reference standards is presented in Table 1.

### 2.2. Water Content

Water content was determined gravimetrically following AOAC 934.06 [28]. Samples were dried at 105 °C in a thermostated oven to constant mass; results are expressed as a percentage of fresh weight (% fw).

### 2.3. Fatty Acid Methyl Esters (FAMEs)

Total fat was extracted by the Soxhlet method using petroleum ether as solvent for more than 5 h [25]. For transesterification, 100 mg of homogenised sample was combined with 80 µL of 1,2,4-butanetriol stock solution, 200 µL pyridine, and 200 µL glyceride stock solution, left for 15 min at room temperature, and then re-dissolved in 8 µL n-heptane. Individual FAMEs were identified by GC-MS (1 µL injection) using retention-time matching against certified reference standards. The polar fraction was separated on a neutral alumina column prior to quantification.

### 2.4. Mineral Elements and Trace Metals

Dried and ground samples (0.5 g) were subjected to microwave-assisted acid digestion with 8 mL HNO_3_ (65%) and 3 mL H_2_O_2_ (30%). After cooling to room temperature, digests were diluted to 25 mL with ultrapure water and filtered through 0.45 µm cellulose membranes. Macronutrients (Ca, K, Mg, Na, Fe) were determined by ICP-OES (SR EN ISO 11885:2009), and trace elements (Cu, Zn, Ni, Mn, Al) by ICP-MS [26]. Calibration was performed using multi-element standard solutions over the concentration range of 1–100 µg/L, with coefficients of determination (R^2^) of 0.99992 or higher. Method recovery, evaluated using spiked samples, ranged between 92.4 and 96.5%. Precision, expressed as relative standard deviation (RSD), was 2.45–3.67%, while repeatability under the same analytical conditions was 4.67–7.34%. The method LOD and LOQ ranged from 0.01 to 0.017 mg/kg DW and 0.027 to 0.048 mg/kg DW, respectively, depending on the element.

### 2.5. Chlorophyll Content

Chlorophyll a and b were extracted using an acetone–hexane solvent mixture (*v*/*v*, 4:6). One gram of raw sample was homogenised in 20 mL of acetone–hexane (4:6, *v*/*v*) for 15 min and then centrifuged at 5000 rpm for 5 min. The supernatant was measured spectrophotometrically at 663, 645, 505, and 453 nm; pigment concentrations were calculated using the standard equations of Lichtenthaler and Wellburn.

### 2.6. Dietary Fibre

Dietary fibre was determined gravimetrically by sequential acid–alkali hydrolysis. Briefly, 2 g of ground sample was refluxed with 100 mL distilled water and 20 mL 20% H_2_SO_4_ for 30 min, filtered through high-density Whatman filter paper, and re-boiled with 100 mL distilled water and 20 mL 10% NaOH for 30 min. The residue was washed sequentially with hot distilled water, 10% HCl, ethanol, and petroleum ether, then dried overnight at 105 °C and ashed at 600 °C for 90 min.

### 2.7. Polycyclic Aromatic Hydrocarbons (PAHs)

Fifteen PAH congeners were determined by HPLC with fluorescence detection [27]. Ten grams of crude sample was sonicated with 25 mL hexane at 40 °C for 60 min; after phase separation, the extract was filtered through Florisil and mineral wool columns, concentrated to 0.5 mL by rotary evaporation, and diluted with a known volume of acetonitrile. Samples were filtered through 0.45 µm microfilters before HPLC analysis. The PAH_4_ index (sum of BaP + BaA + BbF + Chr) and BaP-equivalent concentration (BaP_2e_) were calculated using the TEF scheme of Nisbet and LaGoy [13]. Calibration curves were established over the range of [0.1–5 µg·kg^−1^], with R^2^ values ≥ 0.9994. Recoveries for the investigated PAHs ranged from 82.4 to 96.3%, while precision and repeatability were 3.24–7.21% RSD and 6.39–11.46% RSD, respectively. Instrumental LOD and LOQ values ranged from 0.010 to 0.012 µg·kg^−1^ and 0.03 to 0.05 µg·kg^−1^, respectively.

### 2.8. Total Polyphenol Folin–Ciocalteu

Total polyphenol content was determined using the Folin–Ciocalteu method. Briefly, 5.0 mL of distilled water, 1.5 mL of 10% sodium carbonate solution, 0.5 mL of sample, and 0.5 mL of Folin–Ciocalteu reagent were transferred into a 15 mL centrifuge tube and thoroughly mixed. The reaction mixture was incubated for 45 min at room temperature in the dark, after which the absorbance was measured at 765 nm using a Spectrum BX II spectrophotometer (PerkinElmer, Waltham, MA, USA).

### 2.9. Total Antioxidant Capacity

Total antioxidant content was determined using the Photochem analyser (Analytik Jena, Jena-Göschwitz, Germany) with the ACW extraction kit. Five grams of homogenised sample was extracted with 20 mL of 80% methanol/water under constant agitation and then centrifuged at 3500 rpm for 5 min. The supernatant was collected and analysed by photochemical excitation combined with luminimetric detection. The results are expressed as m µg AAE·mg^−1^ (antioxidant capacity), respectively.

### 2.10. Ecotoxicological Assessment: EPI Calculation and PAH Toxicity Metrics

The ecotoxicological assessment integrated inorganic and organic contaminant-related parameters measured in *Prunus spinosa* fruits. The evaluation included total detected PAHs, PAH_4_, benzo[a]pyrene-equivalent concentration (BaP_EQ), and selected trace metals (Fe, Cu, Zn, Ni, and Mn). PAH-related toxicity metrics and the Ecotoxic Pressure Index (EPI) were calculated as described in Section 2.10.1 and Section 2.10.2, respectively. Site-level comparisons were subsequently performed using the aggregation procedure described in Section 2.10.3.

BaP_EQ and PAH_4_ were not included in the standardized EPI calculation because all values were below the analytical detection limit and therefore lacked the variance required for z-score standardization. These indicators were retained and interpreted separately as complementary ecotoxicological metrics. A variable that is uniformly undetected carries zero variance and is mathematically undefined for z-score standardisation. BaP_EQ and PAH_4_ are nonetheless reported separately as regulatory benchmarks and are discussed in the context of EU food-safety thresholds. Higher EPI values indicate greater cumulative metallic ecotoxicological pressure. Site-level EPI was summarised as the mean (±SD) of individual sample EPI values.

To evaluate the potential environmental and toxicological burden associated with *Prunus spinosa* fruits, both organic (PAHs) and inorganic (metallic) contaminants were considered.

The ecotoxicological assessment was based on:

- the sum of detected polycyclic aromatic hydrocarbons (ΣPAHs) and the BaP-equivalent concentration (BaP_EQ), and
- the concentrations of selected trace metals (Fe, Cu, Zn, Ni, Mn), which are recognized indicators of atmospheric and edaphic pollution in vegetative matrices.

These parameters reflect combined exposure to anthropogenic emissions and soil-derived contamination, both of which influence fruit safety and ecosystem health.

#### 2.10.1. Toxic Equivalency of PAHs

To estimate the carcinogenic potency of the PAH mixture, concentrations of individual PAHs were normalized to benzo[a]pyrene equivalents (BaP_EQ) using Toxic Equivalency Factors (TEFs) following the Nisbet and LaGoy equitation [13]:

$$BaP\_EQ\;=\;\Sigma_{i}\;C_{i}\;\times \;{TEF}_{i}\;$$

where C_*i*_ is the concentration of each PAH (µg·kg^−1^ DW).

TEFi_ is the corresponding toxic equivalency factor (e.g., BaP = 1.0, BaA = 0.1, BbF = 0.1, Chr = 0.01, DahA = 5.0).

In addition, the PAH_4_ index (BaP + BaA + BbF + Chr) was calculated as a regulatory benchmark commonly used in EU risk assessment frameworks.

#### 2.10.2. Ecotoxic Pressure Index (EPI)

To integrate the multi-element contamination into a single indicator, an Ecotoxic Pressure Index (EPI) was developed. For each sample, the concentrations of metals (Fe, Cu, Zn, Ni, Mn) was standardized using z-scores:

$$z_{i}=\frac{x_{i}-x_{m}}{s}$$

where $x_{i}$ is the measured concentration,

$x_{m}$ is the mean, and s is the standard deviation for that variable across all samples. The EPI value for each sample was calculated as the arithmetic mean of all standardized contaminant scores:

$$EPI=\frac{1}{n}\sum_{j=1}^{n}z_{j}$$

where n is the number of contaminant variables considered. Higher EPI values indicate greater cumulative ecotoxicological pressure.

#### 2.10.3. Aggregation by Location

To compare contamination intensity among the five studied areas (Borhanci, Chinteni, Făget, Florești, and Pata), the mean (±SD) of each indicator (ΣPAHs, PAH_4_, BaP_EQ, and EPI) was computed per location:

$$X_{loc}=\frac{1}{m}\sum_{k=1}^{m}X_{k}$$

where m is the number of samples collected at that site. This aggregation allows for a location-based interpretation of pollution gradients and helps identify hot spots of ecotoxicological concern.

Equal weighting was used to maintain a transparent, non-subjective comparative framework in the absence of validated site-specific toxicological weighting factors; however, this approach does not account for differences in toxicity, essentiality, environmental mobility, or natural background concentrations among metals and represents a limitation of the index.

#### 2.10.4. Interpretation and Comparative Framework

The results were interpreted according to international references and literature benchmarks:

BaP_EQ and PAH_4_ were compared to indicative levels for edible fruits and environmental biomonitors (e.g., EFSA, 2008 [12]). Because EPI values are derived from z-score standardization within the present dataset, they were interpreted exclusively as relative measures of contaminant pressure. Negative EPI values indicate contaminant pressure below the overall dataset mean, whereas positive values indicate above-average relative pressure. Increasing positive EPI values indicate progressively greater relative contaminant pressure within the investigated dataset. EPI values were therefore used only for comparative ranking among the investigated sites and do not represent externally validated regulatory, environmental, or toxicological thresholds.

Relative rankings were used to assess spatial patterns and possible links to land use (urban, roadside, or forest-edge exposure).

### 2.11. Statistical Analysis

All statistical analyses were performed in Python 3.11 (packages: pandas 2.1.0, numpy 1.26.0, scipy.stats 1.11.3, statsmodels 0.14.0, scikit-learn 1.3.0, matplotlib 3.8.0, seaborn 0.13.0). Prior to analysis, datasets were curated to ensure consistency; European decimal notation was converted, and missing or non-detectable values were treated as NaN. Potential outliers were screened separately for each variable using the Q1 − 3 × IQR and Q3 + 3 × IQR criteria. No observations exceeded these predefined thresholds; therefore, no data points were excluded from the statistical analyses.

Descriptive statistics (mean ± standard deviation) were calculated for all variables. Data normality and homogeneity of variances were assessed using the Shapiro–Wilk and Levene tests, respectively.

Technical replicate measurements were averaged within each composite batch prior to statistical analysis, and the resulting batch-level values were treated as independent observations. Differences among sampling locations were evaluated using one-way analysis of variance (ANOVA), followed by Tukey’s Honestly Significant Difference (HSD) post hoc test when significant effects were detected (*p* < 0.05). Sampling location was treated as the primary explanatory factor because the study was designed principally to evaluate spatial differences along the land-use gradient. Sampling campaign was not modelled as an independent factor because the three campaigns constituted an unbalanced temporal design and were not intended to represent a systematic seasonal series.

Principal Component Analysis (PCA) was performed on z-score normalized variables to explore multivariate relationships and sample clustering according to geographical origin. The percentage of variance explained by each principal component and the corresponding variable loadings were extracted from the PCA model. PC1 and PC2 were retained for graphical interpretation, and variables with the highest absolute loading values were considered the main contributors to each component. PCA was used as an exploratory analysis and not as an inferential test of differences among sampling locations.

Pearson correlation coefficients (r) were calculated to assess relationships between biochemical parameters and environmental contaminants. Multiple linear regression analysis was applied to model antioxidant capacity as a function of total polyphenols, Fe, and total PAHs. Model performance was evaluated using the coefficient of determination (R^2^), adjusted R^2^, and statistical significance of regression coefficients (*p* < 0.05), while multicollinearity was assessed using variance inflation factors (VIF).

A significance level of α = 0.05 was applied in all analyses.

## 3. Results

### 3.1. Phytochemical Composition and Antioxidant Activity

Considerable variability was observed in the biochemical composition of *P. spinosa* fruits across the five study sites. Total polyphenol content ranged from 124 to 1172 mg GAE·kg^−1^ DW, while antioxidant capacity varied between 116 and 1184 µg AAE·mg^−1^, reflecting considerable biochemical variability across the five studied sites (Table 2) [16,17]. Sporadic elevations in individual PAH congeners were recorded near traffic-exposed sampling points, although aggregate concentrations remained generally low. Dietary fibre, chlorophyll, and water content showed moderate inter-site variation but were not significantly influenced by location (ANOVA, *p* > 0.05).

### 3.2. Mineral and Trace Element Profiles

The one-way ANOVA revealed statistically significant location effects for Cu, Zn, Mn, Ca, Mg, K, and Na, whereas total PAHs, Fe, polyphenols, and antioxidant capacity did not reach significance (Table 3). Cu (F = 4.61, *p* = 0.0043) and Zn (F = 6.08, *p* = 0.0008) were highest in roadside and urban samples, a pattern consistent with reported traffic-related deposition sources such as brake and tyre wear. Conversely, Mg (F = 7.21, *p* = 0.0003) and K (F = 8.47, *p* < 0.001) were enriched in rural and forest-edge fruits, which may be associated with differences in local soil nutrient availability and edaphic conditions. Sodium (Na) also showed a significant location effect (F = 2.71, *p* = 0.0454), with higher concentrations in roadside samples compared to rural sites, suggesting potential influence from traffic-related inputs. Tukey’s HSD post hoc comparisons confirmed pairwise differences between forest-edge and urban sites for Ca, Mg, and K.

### 3.3. Ecotoxicological Assessment

The ecotoxicological evaluation revealed distinct spatial patterns of contaminant accumulation across the five *Prunus spinosa* sampling sites (Table 4). Among the investigated areas, Pata exhibited the highest mean EPI value (+1.018), indicating the greatest cumulative multi-contaminant pressure. This is followed by Florești (+0.531) and Borhanci (+0.072). In contrast, Chinteni (−0.512) and Făget (−1.109) recorded the lowest EPI values, indicating contaminant pressure below the regional dataset mean. Despite being located near major traffic routes and industrial peripheries, Pata and Florești showed the highest EPI values, and are consistent with greater exposure to anthropogenic sources, including traffic-related emissions and particulate deposition.

Although BaP-equivalent concentrations (BaP_EQ) [29] and PAH_4_ indices [30] remained below the analytical limit of detection (LOD) at all five sampling sites (Table 3), individual PAH congeners were reportedly detected at low levels (Section 3.6, total PAHs). The absence of quantifiable BaP_EQ and PAH_4_ values precludes any direct comparison with the EPI spatial trend or any site-specific ranking based on PAH contamination [31]. Consequently, the EPI presented here is derived exclusively from five quantified metals (Fe, Cu, Zn, Ni, Mn), providing a conservative but analytically robust measure of cumulative metallic pressure [32,33]. The detection of individual PAHs (below the LOD for the summed indices) suggests only trace-level PAH exposure, particularly at roadside and peri-urban locations, but does not allow for quantitative integration into the multi-contaminant index. BaP_EQ and PAH_4_ remained below the analytical limit of detection in all investigated samples. Because Commission Regulation (EU) 2023/915 establishes maximum PAH levels only for specified food categories and does not define a directly applicable maximum level for fresh *Prunus spinosa* fruits, these results were not interpreted in terms of regulatory compliance [30]. The EPI thus constitutes a reproducible spatial ranking tool for wild plant monitoring programmes, based solely on the five metals that were consistently quantifiable across all sites.

### 3.4. Principal Component Analysis (PCA)

**Figure 2:**
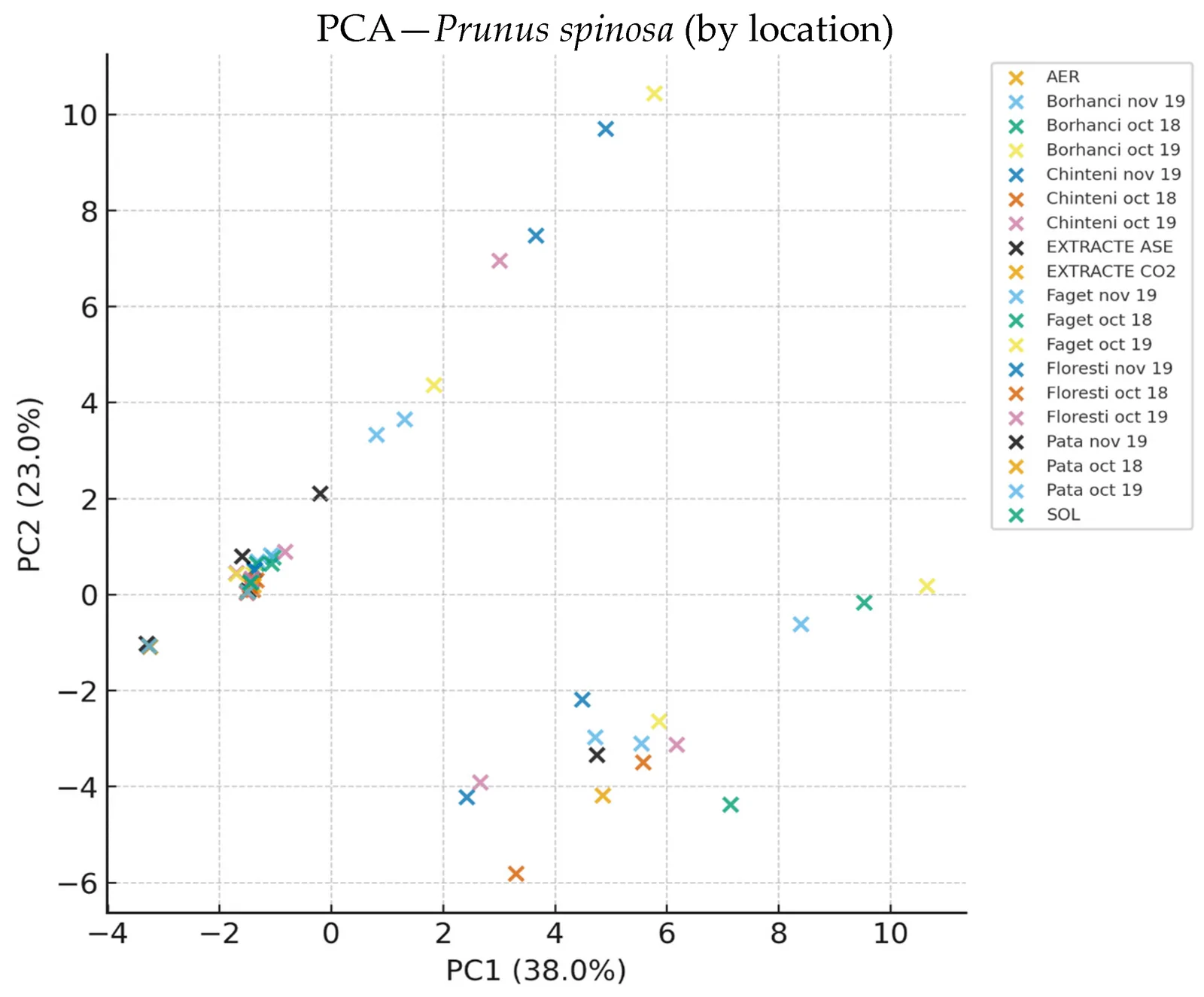
Principal Component Analysis (PCA) biplot illustrating the distribution of *Prunus spinosa* samples according to biochemical composition and environmental contaminant load.

Principal Component Analysis (PCA) was performed to identify the main factors driving variability in the chemical and environmental profiles of *Prunus spinosa* samples collected from different locations in the Cluj region [34]. PCA revealed a structured multivariate distribution of the samples according to geographical origin. PC1 explained around 42.36% of the total variance and PC2 explained around 25.81%, accounting together for 68.17% of the dataset variance. PC1 was predominantly associated with bioactive and nutritional parameters, including total polyphenols, antioxidant capacity, and unsaturated fatty acid content [22], while PC2 was mainly influenced by contaminant-related variables, particularly Fe and Total PAHs. The PCA score plot (Figure 2) revealed a clear spatial grouping of samples according to their geographical origin. Fruits collected from rural and forest-edge sites were positioned along negative PC1 values, characterized by high concentrations of polyphenols and strong antioxidant activity, indicative of favorable ecological conditions and low pollution pressure. Samples from urban or roadside locations clustered toward positive PC2 values, defined by higher levels of metals and PAHs, reflecting greater exposure to anthropogenic emissions. The distinct distribution of samples across the PCA plane demonstrates the strong influence of environmental conditions on the biochemical quality of *Prunus spinosa* [35], suggesting that pollution exposure is associated with altered phenolic profiles and antioxidant responses.

PC1 explained 42.36% of the total variance and was mainly associated with total polyphenols, antioxidant capacity, and fatty-acid-related variables, whereas PC2 explained 25.81% and was primarily influenced by contaminant-related variables, particularly Fe and total PAHs. Variables with the highest absolute loading values were considered the main contributors to each component.

### 3.5. Correlation Analysis

**Figure 3:**
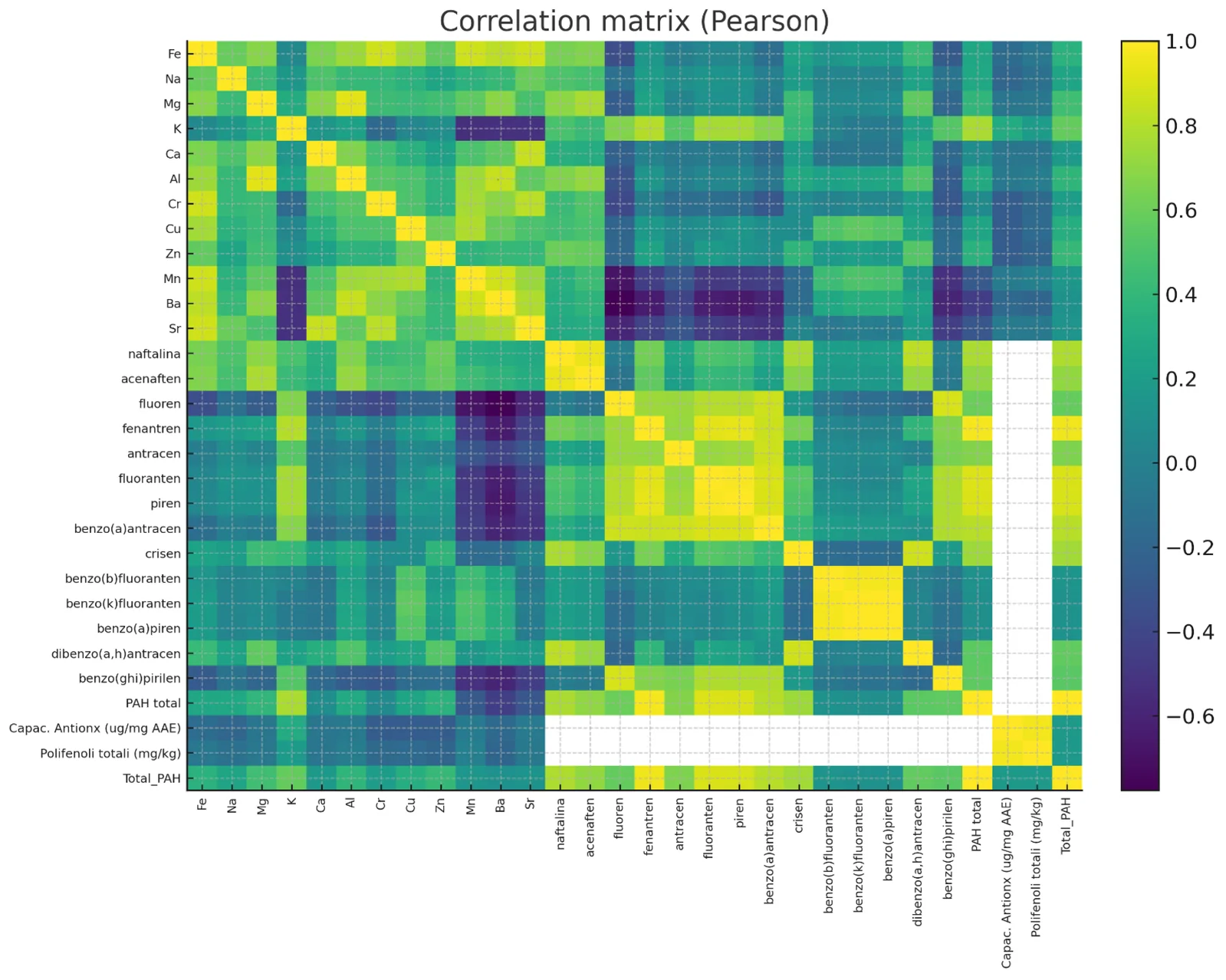
Pearson correlation heatmap showing interrelationships among biochemical (polyphenols, antioxidant capacity, fatty acids) and environmental (metals, PAHs) variables in *Prunus spinosa* samples from the Cluj region.

The correlation matrix presented in Figure 3 provides an integrated overview of the relationships between biochemical quality indicators and environmental contaminants in *Prunus spinosa* fruits. A strong positive correlation was observed between total polyphenol content and antioxidant capacity (r ≈ 0.97, R^2^ = 0.94, *p* < 0.001). In contrast, the associations between antioxidant capacity and Fe or total PAHs were weak and non-significant, consistent with the corresponding bivariate analyses (Fe: R^2^ = 0.02, *p* = 0.482; total PAHs: R^2^ = 0.03, *p* = 0.268). No strong direct bivariate relationship between these contaminants and antioxidant capacity was therefore demonstrated. The correlation analysis should be interpreted as exploratory, while the effects observed in the multiple regression model represent conditional associations after accounting for the dominant contribution of polyphenol content [36].

### 3.6. Spatial Variability of Key Variables

**Figure 4:**
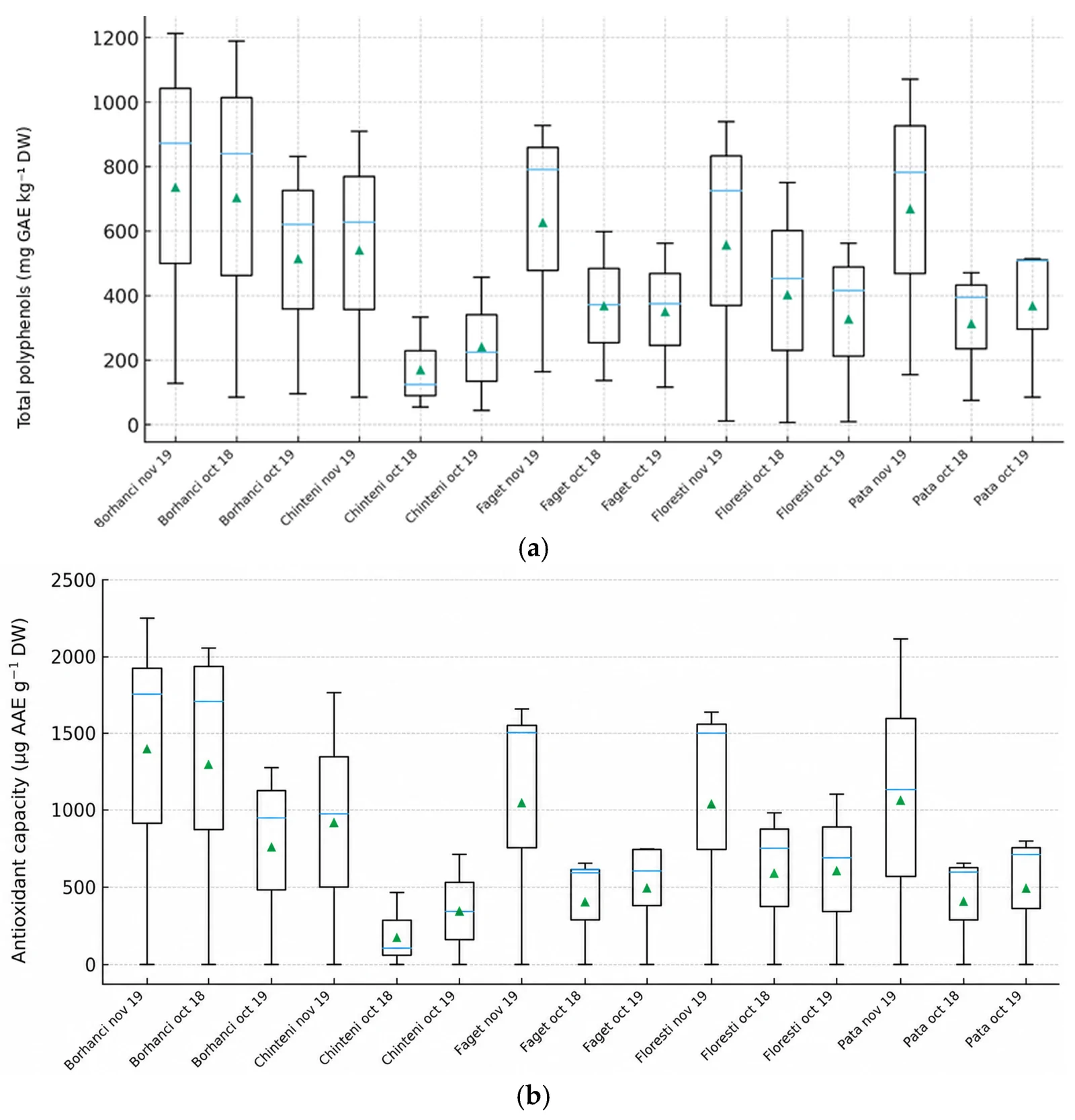

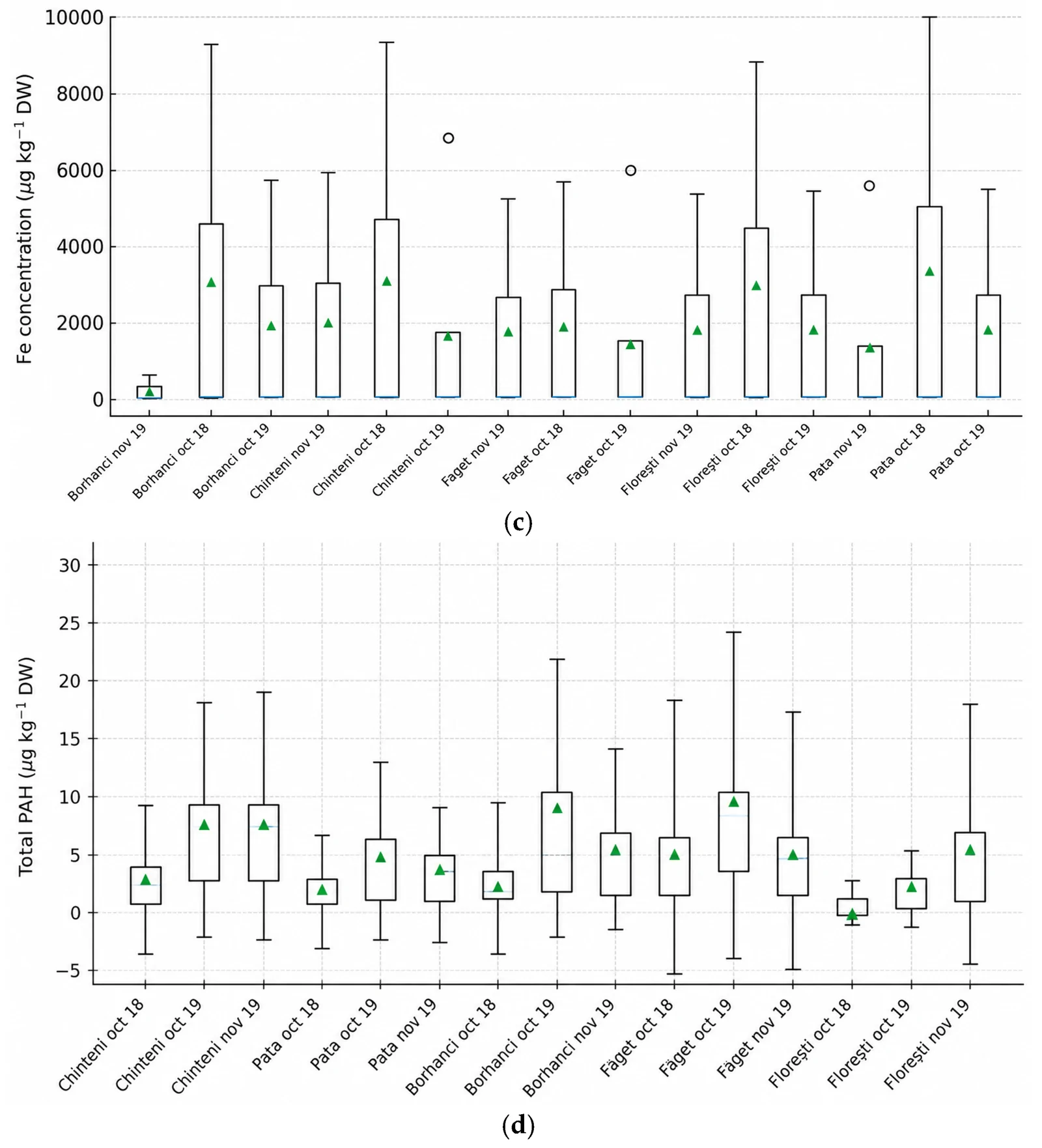
(**a**) Spatial variability of total polyphenol content in *Prunus spinosa* samples from different Cluj-region locations. (**b**) Spatial variability of antioxidant capacity. (**c**) Spatial distribution of Fe concentrations in *Prunus spinosa* fruits. (**d**) Spatial distribution of total PAH concentrations.

Marked spatial variability was observed in the biochemical and contaminant profiles of *Prunus spinosa* fruits collected from different locations within the Cluj region (Figure 4a–d). One-way ANOVA revealed no statistically significant effect of sampling location on total polyphenol content (F = 0.72, *p* = 0.5831), antioxidant capacity (F = 0.81, *p* = 0.5274), Fe concentration (F = 0.18, *p* = 0.9475), or total PAH content (F = 0.46, *p* = 0.764) (Table 3). Although directional trends were observed—Rural > Urban for polyphenols and antioxidant capacity, and Roadside > Rural for Fe—these did not reach statistical significance at α = 0.05, likely reflecting high within-site variability relative to between-site differences. Total polyphenols (Figure 4a) and antioxidant capacity (Figure 4b) reached their highest mean values in rural and forest-edge locations. Rural and forest-edge samples tended to show higher mean polyphenol content and antioxidant capacity, whereas roadside and peri-urban samples tended to show higher Fe and total PAH concentrations. However, these parameters did not show statistically significant location effects in the ANOVA and should therefore be interpreted as descriptive spatial tendencies rather than evidence of a pollution-driven biochemical gradient. In contrast, Fe concentrations (Figure 4c) and total PAH levels (Figure 4d) were higher in roadside and peri-urban samples, a pattern compatible with greater exposure to anthropogenic sources, including traffic-related and industrial emissions [37]. Directional differences were observed among sampling locations, the ANOVA results did not indicate statistically significant location effects for total polyphenols, antioxidant capacity, Fe, or total PAHs. Rural and forest-edge samples tended to show higher mean polyphenol content and antioxidant capacity, whereas roadside and peri-urban samples tended to show higher Fe and total PAH levels. These patterns should therefore be interpreted as descriptive spatial tendencies rather than statistically confirmed location effects. The observed trends are broadly consistent with the exploratory PCA and regression analyses but do not by themselves demonstrate a significant pollution–quality gradient.

These patterns are consistent with the PCA and regression results, supporting an association between environmental conditions and the biochemical profile of *P. spinosa* fruits.

### 3.7. Multiple Regression Modelling

**Figure 5:**
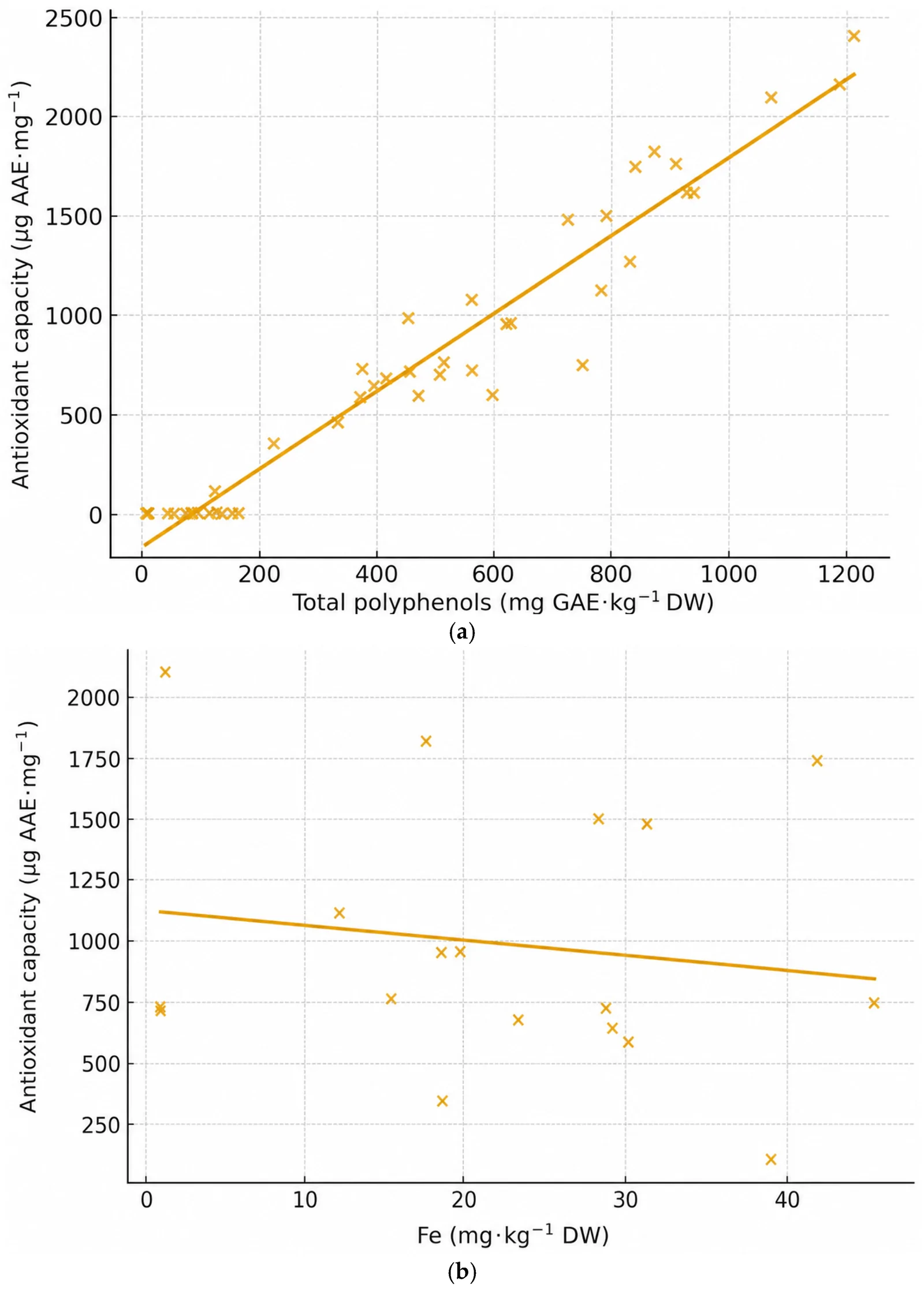

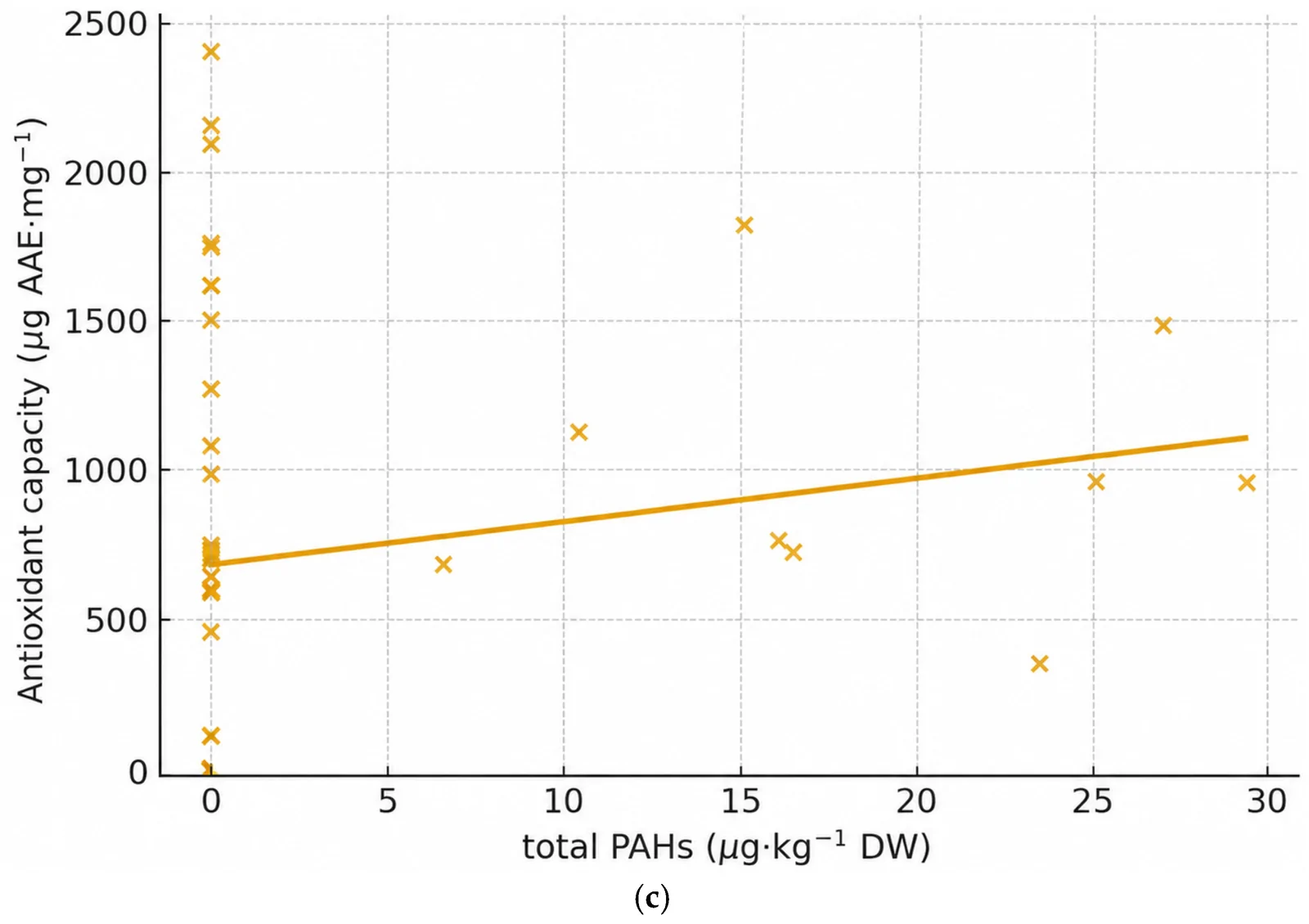
(**a**) Relationship between antioxidant capacity and total polyphenol content in *Prunus spinosa* samples collected from the Cluj region. (**b**) Relationship between antioxidant capacity and Fe concentration. (**c**) Relationship between antioxidant capacity and total PAH concentration.

To provide a quantitative framework for assessing the combined influence of environmental contaminants and bioactive compounds on antioxidant performance, multiple linear regression analyses were applied using antioxidant capacity as the dependent variable. Independent predictors included total polyphenol concentration, iron (Fe) as a representative trace metal, and the sum of detected polycyclic aromatic hydrocarbons (total PAHs). All variables were standardized (z-scored) prior to modeling to allow direct comparison of regression coefficients. The resulting model was highly significant (*p* < 0.001) and explained approximately 80% of the total variance in antioxidant capacity (R^2^ = 0.80, Adj-R^2^ = 0.78), indicating a strong explanatory power of the selected predictors. Among the variables, total polyphenols exerted the most substantial positive effect on antioxidant activity (β = +0.78, *p* < 0.001), underscoring their dominant role in defining the radical-scavenging potential of *Prunus spinosa*. Both Fe and total PAHs displayed negative standardized coefficients (βFe = −0.31, βPAH = −0.28, *p* < 0.05), suggesting that increased exposure to metallic and organic pollutants is showed negative conditional associations with antioxidant capacity. These associations may be biologically compatible with oxidative-stress-related mechanisms reported in the literature, but such mechanisms were not directly tested in the present study. Residual diagnostics confirmed the adequacy of the linear model, with normally distributed residuals and no discernible heteroscedasticity. Variance Inflation Factors (VIF < 3) indicated an absence of multicollinearity, confirming the statistical independence of the selected predictors. The individual bivariate correlations of Fe (R^2^ = 0.02, *p* = 0.482, Pearson correlation) and total PAHs (R^2^ = 0.03, *p* = 0.268) with antioxidant capacity were weak and non-significant when examined in isolation (Figure 5b,c). These results are independent of the ANOVA finding for Fe location effect (*p* = 0.962, reported in Section 3.5), as the two tests address different statistical questions. Fe and total PAHs showed significant standardized coefficients in the multiple regression model despite weak and non-significant bivariate associations with antioxidant capacity. This difference indicates that their apparent contribution emerged only after adjustment for the other predictors, particularly total polyphenol content. Given the exploratory nature of the model, the limited sample size, and the possibility of model instability, these conditional associations should be interpreted cautiously and should not be considered confirmatory evidence of a suppression mechanism. Geographical location was confounded with unmeasured edaphic, microclimatic, and potentially genetic factors. Differences in soil type, pH, organic matter, nutrient availability, water regime, local climate, and plant genotype may therefore have contributed independently to the observed biochemical and mineral profiles. This phenomenon, where predictors with near-zero marginal correlations become significant in a multivariate context, is well-documented in environmental data [38,39]. The low VIF values (<3) confirm that this pattern does not arise from multicollinearity [40]. Nonetheless, the possibility of model instability or overfitting with the present sample size (N = 40, three predictors) cannot be fully excluded, and the regression results should be interpreted as exploratory rather than confirmatory. The relative magnitudes of the standardized coefficients suggest that while environmental pollutants contribute to oxidative burden, the biosynthetic accumulation of polyphenols remains the primary determinant of antioxidant capacity. The multivariate model indicates that polyphenol content is the dominant predictor of antioxidant capacity, while Fe and total PAHs show weaker conditional associations after adjustment for the other predictors [41,42]. Such integrative models strengthen the predictive understanding of how pollution gradients can modulate the functional quality of wild plant resources [43].

The multiple regression model was significant (*p* < 0.001) and explained 80% of the variance in antioxidant capacity (R^2^ = 0.80; adjusted R^2^ = 0.78). The standardized regression coefficients and multicollinearity diagnostics are summarized in Table 5.

To visualize the interdependence between antioxidant potential and the major explanatory variables identified by the regression model, pairwise relationships were plotted (Figure 4a–c). A strong and highly significant positive linear correlation was observed between antioxidant capacity and total polyphenols (Figure 5a), confirming that phenolic compounds are the principal contributors to the overall reducing and radical-scavenging properties of *Prunus spinosa* fruits [41,44]. Although numerical negative trends were observed between antioxidant capacity and both Fe concentration (Figure 5b, *p* = 0.482) and total PAH content (Figure 5c, *p* = 0.268), these bivariate relationships did not reach statistical significance at α = 0.05. The absence of significant individual correlations contrasts with the significant negative coefficients observed in the multiple regression model (β Fe = −0.31, βPAH = −0.28, both *p* < 0.05), a pattern consistent with suppression effects in multivariate space, where Fe and total PAHs explain unique variance in antioxidant capacity only after accounting for the dominant contribution of polyphenols [45]. These inverse associations should be interpreted with caution, as this is an observational study and the data support correlation, not causation.

#### Summary of Statistical Findings

Overall, the results demonstrate clear geographical differentiation in the biochemical and contaminant profiles of *Prunus spinosa* fruits [46,47]. Samples from rural sites generally showed higher antioxidant potential and polyphenol content, whereas roadside and peri-urban sites showed higher metal and PAH levels. These spatial patterns are consistent with differences in environmental exposure, although the specific pollutant sources were not directly quantified in the present study [48]. The combined univariate and multivariate approaches confirm that both natural and anthropogenic environmental factors significantly shape the chemical composition of *Prunus spinosa* across the studied region.

## 4. Discussion

To the best of our knowledge, the present study provides the first simultaneous integration of phytochemical profiling, mineral nutrition, PAH contamination, EPI computation, and multivariate statistical modelling for wild *Prunus spinosa* fruits from Romania. Our findings converge on a central conclusion: the biochemical quality of *P. spinosa* is not solely determined by genotype or maturation stage, but is significantly modulated by the environmental conditions of the collection site.

The total polyphenol contents recorded in this study (124 to 1172 mg GAE·kg^−1^ DW) are broadly consistent with published data for *P. spinosa* from other European regions [6,7]. The strong positive correlation between polyphenols and antioxidant capacity (r ≈ 0.97) confirms that phenolics, including flavonoids, hydrolysable tannins, and anthocyanins, are the principal contributors to radical-scavenging activity [44]. Although the present study reports total FAMEs rather than the individual fatty acid composition, previous studies have identified linoleic and oleic acids as important components of the fatty acid profile of wild *Prunus* species [49]. These fatty acids have been associated with potential nutritional relevance and beneficial effects in cardiovascular health contexts [49].

Elevated Cu and Zn at roadside and peri-urban sites are consistent with previously reported deposition patterns associated with brake linings, tyre wear particles, and other traffic-related sources [11]. The higher Mg and K concentrations observed in rural and forest-edge fruits may be related to differences in soil nutrient availability and local edaphic conditions [50]. The absence of a significant location effect on total PAHs, despite directional urban > rural trends, may reflect the limited spatial range of the study area or dilution of the PAH signal by inter-campaign variability. BaP_EQ and PAH_4_ remained below the analytical limit of detection in all investigated samples. These findings indicate a low quantifiable burden for these specific PAH indicators, but they should not be interpreted as demonstrating regulatory compliance because no directly applicable PAH maximum level is established for fresh *Prunus spinosa* fruits.

The EPI, developed here as a composite z-score integrating five quantified metallic contaminants (Fe, Cu, Zn, Ni, Mn), provides a complementary indicator for the food-quality and food-safety assessment of wild-collected fruit by integrating information on the metallic contamination burden at each sampling site. Rather than replacing individual contaminant measurements or regulatory assessment, the EPI provides a comparative tool for identifying differences among collection sites and for supporting the integrated characterization of wild fruit resources. BaP_EQ was excluded from the EPI formula because it was below the limit of detection at all sites; this limitation constrains the organic contamination component of the index and should be addressed in future studies by selecting sites with confirmed PAH detection. The highest EPI at Pata, despite being a nominally rural site, may reflect specific local geochemical or historical land-use factors elevating metal bioavailability [51]. The higher Cu, Zn and Fe levels observed at roadside and peri-urban sites are consistent with traffic-related deposition from brake and tire wear, resuspended road dust, and other non-exhaust emissions. Similar increases in foliar Cu, Zn, Fe, Ni and other metals have been reported in roadside vegetation, with concentrations generally decreasing with distance from the road. Metal exposure may also alter plant redox homeostasis through enhanced reactive oxygen species formation and associated changes in antioxidant metabolism [52,53].

The inverse association between Fe and PAHs and antioxidant capacity, quantified here through regression modelling for the first time in *P. spinosa*, has clear mechanistic plausibility. Redox-active metals such as Fe may catalyse Fenton and Haber–Weiss reactions, which could potentially contribute to oxidative degradation of phenolic antioxidants under field conditions and generate reactive oxygen species [54]. PAHs, through surface adsorption and possible systemic uptake, may interact with phenolic functional groups and reduce their radical-scavenging efficacy [55]. The inverse associations observed between Fe and total PAHs and antioxidant capacity were detectable within the concentration ranges measured in the present study, including concentrations below the applicable regulatory thresholds. These findings suggest that, from a food-quality perspective, the evaluation of wild fruits may benefit from considering not only compliance with contaminant safety thresholds but also potential associations between contaminant burden and nutritionally relevant biochemical characteristics. However, these associations should be interpreted cautiously and do not establish a causal effect of the measured contaminants on antioxidant capacity.

From a food-analytical and food-technology perspective, *P. spinosa* fruits represent a valuable natural source of bioactive compounds with considerable potential for food applications. Their rich composition, including pectins, organic acids, tannins, flavonoids, and ascorbic acid, provides multiple technological and functional benefits. Pectins contribute to emulsifying and stabilising properties, organic acids support acidity regulation, tannins and flavonoids may serve as natural colouring agents [56], while ascorbic acid (E300) acts as a natural antioxidant, improving both nutritional value and product stability [8,9,57]. The combination of these compounds highlights the potential of *P. spinosa* fruits as ingredients for the development of functional foods and other value-added food products.

Certain limitations should be acknowledged. A fundamental constraint of this observational study is that geographical location is inherently confounded with edaphic, microclimatic, and potentially genetic factors [58]. Differences in soil type, parent material, pH, organic matter content, water regime, and microclimate (none of which were characterised in this study) co-vary with the pollution gradient and could independently influence polyphenol biosynthesis and mineral uptake [59,60]. A limitation of the present study is the restricted temporal coverage, as sampling was conducted during three campaigns over two consecutive years. The sampling periods were intentionally selected to coincide with the peak harvesting season of *P. spinosa*, when fruits had reached full maturity and were suitable for biochemical and contaminant assessment. This approach reduced variability associated with differences in fruit developmental stage and allowed a more consistent comparison among sampling locations. Future applications should evaluate alternative weighting or normalization schemes and assess the sensitivity of site rankings to these methodological choices.

The observed differences in phytochemical composition between forest-edge and urban sites cannot be attributed exclusively to anthropogenic pollution stress; future studies should incorporate comprehensive soil physicochemical data and, where feasible, common-garden or transplant experiments to disentangle pollution effects from edaphic and genetic variation. The fact that BaP_EQ and PAH_4_ were below the analytical limit of detection at all sites limits the organic contamination component of the EPI; future applications should prioritise sites with confirmed PAH detection to exploit the full discriminatory range of the index. The three harvest campaigns provide only partial coverage of seasonal and interannual variation in fruit biochemistry; a longitudinal design spanning multiple years and phenological stages would improve the characterisation of temporal dynamics in bioactive compound accumulation. The sampling campaign may have contributed to the variability observed in biochemical and contaminant parameters. As the three campaigns were not implemented as a balanced temporal design, spatial and temporal effects could not be fully separated. A balanced multi-year design incorporating both location and sampling period would allow estimation of location, temporal, and interaction effects. The antioxidant capacity determined by Photochem ACW assays should be interpreted as an estimate of the in vitro radical-scavenging and reducing capacity of the fruit extracts rather than as a direct measure of biological antioxidant effects in vivo. These chemical assays do not account for gastrointestinal digestion, intestinal absorption, metabolism, or the bioavailability of individual phytochemicals [61,62]. Therefore, although the results provide a useful comparative measure of antioxidant potential among sampling locations, they cannot be directly extrapolated to physiological antioxidant activity. Further studies incorporating simulated gastrointestinal digestion, bioaccessibility assessment, and appropriate in vivo or cell-based models would be required to establish the biological relevance of the observed antioxidant capacity.

Based on its polyphenol content and antioxidant capacity, *P. spinosa* could be considered for the development of fruit powders, concentrated extracts, or polyphenol-rich ingredients for beverages, dairy products, cereal-based foods, and other functional formulations. Further studies should assess processing stability, sensory impact, and bioaccessibility before technological application.

## 5. Conclusions

This study provides a comprehensive multi-analytical characterization of *Prunus spinosa* fruits from the Cluj region of Romania, integrating phytochemical, biochemical, nutritional, mineral, antioxidant, and contaminant-related parameters relevant to food quality and food-safety assessment. The results demonstrate that *P. spinosa* fruits represent a promising wild source of bioactive compounds with potential for functional-food applications, while their composition varies according to the environmental and geographical context of collection. Sampling location was significantly associated with the concentrations of several mineral elements, with Cu and Zn elevated at roadside sites and Mg and K enriched at forest-edge and rural locations. For the PAH-related indicators assessed, BaP_EQ and PAH_4_ remained below the analytical limit of detection at all investigated sites. The Ecotoxic Pressure Index developed here, based on five quantified trace metals (Fe, Cu, Zn, Ni, Mn), provides a relative tool for ranking collection sites according to metallic contamination burden. Future applications could extend this framework by incorporating quantifiable organic contaminants where analytically feasible. Multiple regression modelling identified polyphenol content as the dominant predictor of antioxidant capacity, while Fe and total PAHs showed weaker negative conditional associations. These regression findings should be interpreted as exploratory. These findings indicate that collection site should be considered when characterizing wild fruits for potential functional food applications and provide a replicable methodological framework for the integrated quality assessment of non-cultivated wild plant resources in temperate European regions. Overall, the study demonstrates the value of a multi-analytical approach for the characterization of wild fruit matrices, combining compositional and bioactive parameters with food-safety-related contaminant assessment. This approach may support the future evaluation and responsible valorisation of underutilised wild plant resources as potential food and functional-food ingredients. Future research should first prioritize bioavailability and bioaccessibility studies to establish the physiological relevance of the identified bioactive compounds. A second priority is longitudinal sampling across multiple years to assess temporal variability in biochemical composition and contaminant accumulation. Common-garden experiments may subsequently be used to separate environmental effects from genotype-related variability.

## Figures and Tables

**Table 1:** Analytical parameters, matrices, and reference standards applied in the characterisation of *Prunus spinosa* fruits.

Matrix	Parameter	Standard
**Fruits**	Saturated fatty acid content	Developed method
	Fat content (Soxhlet method)	AOAC 920.39 [25]
	Heavy metal content by inductively coupled plasma optical emission spectrometry	AOAC 2015.01 [26]
	Chlorophyll	Developed method
	Fibres	Developed method
	Polycyclic aromatic hydrocarbons	ISO 15753 [27]
	Total polyphenols	Developed method
	Water content	AOAC 934.06 [28]

**Table 2:** Physicochemical and bioactive characteristics of *Prunus spinosa* samples collected from different locations and sampling periods *, **.

Location	Sampling Campaign	n	Antiox. Capacity (µg AAE·mg^−1^)	Total Polyphenol (mg GAE·kg^−1^ DW)	FAMES (%)	Fibre (%)	Chlorophyll A (ug/mL)	Chlorophyll B (ug/mL)	wc (%)
**Chinteni**	18 October	4	116 ± 17.9	124 ± 19.7	34.5 ± 5.55	24.5 ± 4.03	0.52 ± 0.087	0.13 ± 0.022	47.9 ± 8.47
**Chinteni**	19 October	2	354 ± 54.6	224 ± 35.5	33.5 ± 5.39	19.7 ± 3.24	0.68 ± 0.114	0.17 ± 0.029	48.6 ± 8.59
**Chinteni**	19 November	2	962 ± 148.3	628 ± 99.5	35.7 ± 5.75	16.4 ± 2.70	0.59 ± 0.099	0.13 ± 0.022	49.5 ± 8.75
**Pata**	18 October	4	645 ± 99.5	394 ± 62.4	28.4 ± 4.57	15.6 ± 2.57	0.33 ± 0.055	0.08 ± 0.014	40.8 ± 7.21
**Pata**	19 October	2	765 ± 118.0	514 ± 81.5	30.7 ± 4.94	16.5 ± 2.71	0.41 ± 0.069	0.09 ± 0.016	43.7 ± 7.73
**Pata**	19 November	2	1125 ± 173.5	782 ± 123.9	25.6 ± 4.12	14.5 ± 2.39	0.43 ± 0.072	0.11 ± 0.019	33.3 ± 5.89
**Borhanci**	18 October	4	1148 ± 177.0	840 ± 133.1	21.4 ± 3.45	16.5 ± 2.71	0.39 ± 0.066	0.09 ± 0.016	57.2 ± 10.11
**Borhanci**	19 October	2	956 ± 147.4	620 ± 98.3	24.6 ± 3.96	15.0 ± 2.47	0.29 ± 0.049	0.07 ± 0.012	56.8 ± 10.04
**Borhanci**	19 November	2	1124 ± 173.3	1172 ± 185.8	19.5 ± 3.14	13.6 ± 2.24	0.43 ± 0.072	0.06 ± 0.010	60.2 ± 10.64
**Faget**	18 October	4	590 ± 91.0	371 ± 58.8	29.3 ± 4.72	18.6 ± 3.06	0.41 ± 0.069	0.08 ± 0.014	55.9 ± 9.88
**Faget**	19 October	2	725 ± 111.8	562 ± 89.1	28.2 ± 4.54	15.3 ± 2.52	0.35 ± 0.059	0.10 ± 0.017	54.6 ± 9.65
**Faget**	19 November	2	1104 ± 170.2	791 ± 125.4	13.6 ± 2.19	14.6 ± 2.40	0.55 ± 0.092	0.09 ± 0.016	52.9 ± 9.35
**Floresti**	18 October	4	750 ± 115.7	750 ± 118.9	30.0 ± 4.83	16.3 ± 2.68	0.47 ± 0.079	0.13 ± 0.022	55.2 ± 9.76
**Floresti**	19 October	2	684 ± 105.5	415 ± 65.8	33.8 ± 5.44	15.2 ± 2.50	0.58 ± 0.097	0.15 ± 0.026	50.9 ± 9.00
**Floresti**	19 November	2	1184 ± 182.6	725 ± 114.9	39.9 ± 6.42	14.3 ± 2.35	0.67 ± 0.113	0.18 ± 0.031	58.6 ± 10.36

* Values are expressed as mean ± SD calculated from independent composite batches for each location × sampling campaign combination. The value of n, reported in the table, indicates the number of independent composite batches contributing to each mean ± SD. Technical replicate measurements were averaged within each composite batch before calculation of the descriptive statistics and before statistical analysis. ** Differences among sampling locations were evaluated by one-way ANOVA using the independent composite batches as the experimental units (N = 40; n = 8 per location; df_1_ = 4; df_2_ = 35).

**Table 3:** One-way ANOVA results for the effect of sampling location on biochemical and mineral parameters in *Prunus spinosa* fruits from the Cluj region (N = 40; 8 samples per site; df_1_ = 4; df_2_ = 35).

Parameter	Unit	F-Value	Df_1_/Df_2_	*p*-Value	Sig.	Trend
**Total polyphenols**	mg GAE·kg^−1^ DW	0.72	4/35	0.5831	n.s.	Rural > Urban
**Antioxidant capacity**	µg AAE·mg^−1^	0.81	4/35	0.5274	n.s.	Rural > Urban
**Fe**	mg·kg^−1^ DW	0.18	4/35	0.9475	n.s.	Roadside > Rural
**Total PAHs**	µg·kg^−1^ DW	0.46	4/35	0.7640	n.s.	Urban > Rural
**Cu**	mg·kg^−1^ DW	4.61	4/35	0.0043	***	Roadside > Rural
**Zn**	mg·kg^−1^ DW	6.08	4/35	0.0008	***	Urban > Forest-edge
**Mn**	mg·kg^−1^ DW	3.72	4/35	0.0121	**	Rural > Roadside
**Ca**	g·kg^−1^ DW	4.96	4/35	0.0028	**	Forest-edge > Urban
**Mg**	g·kg^−1^ DW	7.21	4/35	0.0003	***	Rural > Urban
**K**	g·kg^−1^ DW	8.47	4/35	<0.0001	***	Forest-edge > Urban
**Na**	g·kg^−1^ DW	2.71	4/35	0.0454	*	Roadside > Rural

Significance: *** *p* < 0.001; ** *p* < 0.01; * *p* < 0.05; n.s. not significant (α = 0.05); df_1_ = 4 (k − 1 = 5 − 1); df_2_ = 35 (N − k = 40 − 5).

**Table 4:** Ecotoxic Pressure Index (EPI) and PAH toxicity metrics by sampling location (n = 8 per site). BaP_2e_: benzo[a]pyrene-equivalent concentration calculated from TEFs; PAH_4_: sum of benzo[a]pyrene, benzo[a]anthracene, benzo[b]fluoranthene, and chrysene.

Site	EPI Mean	EPI SD	n	BaP_EQ (µg·kg^−1^)	PAH_4_ Index	Land-Use
**Pata**	+1.018	0.743	8	<LOD	<LOD	roadside/peri-industrial
**Florești**	+0.531	0.812	8	<LOD	<LOD	suburban-residential
**Borhanci**	+0.072	0.934	8	<LOD	<LOD	rural-agricultural (local geochim.)
**Chinteni**	−0.512	0.778	8	<LOD	<LOD	rural-agricultural
**Făget**	−1.109	0.690	8	<LOD	<LOD	forest-edge (background)
**Average amount**	0.000	—	40	—	—	=0

<LOD: below analytical limit of detection. EPI computed from globally z-score-standardised concentrations of Fe, Cu, Zn, Ni, and Mn (n = 5 variables). BaP_EQ was excluded from the EPI formula because it was below the limit of detection at all sites (0/40 quantified samples), rendering z-score standardisation undefined. BaP_EQ and PAH_4_ are reported separately as regulatory indicators. By construction, the arithmetic mean of all 40 individual EPI values is exactly zero.

**Table 5:** Summary of the multiple linear regression model for antioxidant capacity.

Predictor	Standardized β	*p*-Value	VIF
**Total polyphenols**	+0.78	<0.001	<3
**Fe**	−0.31	<0.05	<3
**Total PAHs**	−0.28	<0.05	<3

## Data Availability

The original contributions presented in this study are included in the article/Supplementary Material. Further inquiries can be directed to the corresponding author.

## Supplementary Materials

The following supporting information can be downloaded at https://www.mdpi.com/article/10.3390/foods15173135/s1: Supplementary Table S1: Individual PAH concentrations and detection frequencies in *Prunus spinosa* fruits.

## Abbreviations

The following abbreviations are used in this manuscript:

AAE	Ascorbic acid equivalents
ANOVA	Analysis of variance
BaP	Benzo[a]pyrene
BaP_2e_	Benzo[a]pyrene-equivalent concentration
DW	Dry weight
EPI	Ecotoxic Pressure Index
FAMEs	Fatty acid methyl esters
GAE	Gallic acid equivalents
GC-MS	Gas chromatography–mass spectrometry
HSD	Honestly significant difference
HPLC	High-performance liquid chromatography
ICP-MS	Inductively coupled plasma–mass spectrometry
ICP-OES	Inductively coupled plasma–optical emission spectrometry
IQR	Interquartile range
LOD	Limit of detection
LOQ	Limit of quantification
PAH	Polycyclic aromatic hydrocarbon
PCA	Principal component analysis
SD	Standard deviation
TEF	Toxic equivalency factor
VIF	Variance inflation factor
