# Supplementary material for: Biochemical Profiling and Ecotoxicological Assessment of Prunus spinosa L. Fruits from the Cluj Region: Phytochemical Composition, Contaminant Load, and Antioxidant Potential
Source: Foods. 2026 Sep 3;15(17):3135. doi: 10.3390/foods15173135 (PMC13564682; doi:10.3390/foods15173135)
Supplement: Supplementary file 1 [file foods-15-03135-s001.zip › foods-4514725-supplementary.pdf]

Supplementary Table S1. Individual PAH concentrations and detection frequencies in *Prunus spinosa* fruits

**PAH congener**	**Detected concentration range (µg·kg^{-1} DW)**	**Method LOD (µg·kg^{-1})**	**Method LOQ (µg·kg^{-1})**	**Detection frequency**
Naphthalene	0.23–0.90	0.010–0.012*	0.03–0.05*	(100%)
Acenaphthene	0.36–3.81	0.010–0.012*	0.03–0.05*	(100%)
Fluorene	3.71–13.40	0.010–0.012*	0.03–0.05*	(100%)
Phenanthrene	0.13–1.38	0.010–0.012*	0.03–0.05*	(88.9%)
Anthracene	0.43–2.85	0.010–0.012*	0.03–0.05*	(100%)
Fluoranthene	0.68–2.72	0.010–0.012*	0.03–0.05*	(100%)
Pyrene	0.14–1.77	0.010–0.012*	0.03–0.05*	(100%)
Benzo[a]anthracene	0.18–0.53	0.010–0.012*	0.03–0.05*	(77.8%)
Chrysene	Not detected	0.010–0.012*	0.03–0.05*	(0%)
Benzo[b]fluoranthene	Not detected	0.010–0.012*	0.03–0.05*	(0%)
Benzo[k]fluoranthene	Not detected	0.010–0.012*	0.03–0.05*	(0%)
Benzo[a]pyrene	Not detected	0.010–0.012*	0.03–0.05*	(0%)
Dibenzo[a,h]anthracene	0.28–0.92	0.010–0.012*	0.03–0.05*	(62.5%)
Benzo[g,h,i]perylene	6.43–27.10	0.010–0.012*	0.03–0.05*	(100%)
Indeno (1,2, 3 cd)pyren	Not detected	0.010–0.012*	0.03–0.05*	(0%)

*The manuscript currently reports the **overall method-specific LOD and LOQ ranges across the investigated PAHs**, not individual congener-specific values.
